# White Matter and Cognition in Adults Who Were Born Preterm

**DOI:** 10.1371/journal.pone.0024525

**Published:** 2011-10-12

**Authors:** Matthew P. G. Allin, Dimitris Kontis, Muriel Walshe, John Wyatt, Gareth J. Barker, Richard A. A. Kanaan, Philip McGuire, Larry Rifkin, Robin M. Murray, Chiara Nosarti

**Affiliations:** 1 King's Health Partners, King's College London, Department of Psychosis Studies, Institute of Psychiatry, London, United Kingdom; 2 Department of Neonatal Paediatrics, Royal Free & University College Medical School, London, United Kingdom; City of Hope National Medical Center and Beckman Research Institute, United States of America

## Abstract

**Background and Purpose:**

Individuals born very preterm (before 33 weeks of gestation, VPT) are at risk of damage to developing white matter, which may affect later cognition and behaviour.

**Methods:**

We used diffusion tensor MRI (DT-MRI) to assess white matter microstructure (fractional anisotropy; FA) in 80 VPT and 41 term-born individuals (mean age 19.1 years, range 17–22, and 18.5 years, range17–22 years, respectively). VPT individuals were part of a 1982–1984 birth cohort which had been followed up since birth; term individuals were recruited by local press advertisement. General intellectual function, executive function and memory were assessed.

**Results:**

The VPT group had reduced FA in four clusters, and increased FA in four clusters relative to the Term group, involving several association tracts of both hemispheres. Clusters of increased FA were associated with more severe neonatal brain injury in the VPT group. Clusters of reduced FA were associated with lower birth weight and perinatal hypoxia, and with reduced adult cognitive performance in the VPT group only.

**Conclusions:**

Alterations of white matter microstructure persist into adulthood in VPT individuals and are associated with cognitive function.

## Introduction

Preterm birth is associated with a range of adverse outcomes, including impaired cognitive function and academic underperformance [1]–[3]. These sequelae have been attributed to perinatal brain injury [4], particularly when this involves white matter [5]–[11]. White matter may be susceptible to damage after preterm birth because of the developmental vulnerability of oligodendrocyte precursors [12], [13]. A recent DT-MRI study [71] reported that the pattern of diffusivity abnormalities in extremely preterm neonates suggested an oligodendroglial rather than axonal lesion. White matter volume has been shown to be reduced in preterm adolescents [10], [14]. Diffusion tensor magnetic resonance imaging (DT-MRI) provides information about the microstructure of white matter. The principle DT-MRI measure is fractional anisotropy (FA), which describes the degree to which water diffusion is constrained in a certain direction [15], and is likely to be sensitive to both the alignment of white matter fibres and their structural integrity, including the degree of myelination [16]–[18]. FA is correlated with age [19], [20], and with cognitive maturation [21]–[23]. DT-MRI studies indicate that white matter microstructure is altered in preterm neonates [24]–[26] and children [21] and in very-low-birth-weight adolescents [29], [30] and is associated with neurocognitive outcome [27]–[30]. It has been suggested that DT-MRI indices may be a useful indicator of individuals at greatest risk of poor developmental outcomes [72]. Few studies have followed preterm-born individuals into adulthood [11], [31], [32], and it is at present unclear to what extent white matter abnormalities may be attenuated by brain growth and maturation.

In this study we assessed a group of young adults born before 33 weeks of gestation (very preterm; VPT) and a Term-born comparison group using DT-MRI and neuropsychological evaluation. We hypothesised that the VPT group would show altered patterns of white matter microstructure, and that these would be associated with perinatal adversity and with adult cognitive function.

## Methods

### Study Participants

VPT individuals were recruited from a cohort born before 33 weeks of gestation between 1982 and 1984 and admitted to University College London Hospital within five days of birth. Three hundred and two individuals survived and were recruited into the study [7], [8]. At 15 years, 111 individuals were assessed [7]. These individuals were re-contacted and 87 (78%) underwent DT-MRI, at mean age 19.1 years (range 17–22 years). VPT individuals who were not assessed did not differ significantly from those who were assessed in their gestational age (t = 1.55, df = 469, p = 0.12), Apgar scores at 1 minute (χ2 = 0.3, df = 1, p = 0.86) and 5 minutes (χ2 = 0.9, df = 1, p = 0.35), gender (χ2 = 0.82, df = 1, p = 0.37), or socioeconomic status (SES), determined according to the Registrar General's (1991) classification [67], (χ2 = 1.3, df = 4, p = 0.87).

Forty-nine term-born individuals were recruited by advertisement in the South London press at a mean age of 18.5 years (range 17–22 years). They were screened for the presence of neurological conditions and a history of intracranial infection or head injury.

### Neuropsychological Testing

VPT and Term participants were assessed, blind to group membership, using the Wechsler Abbreviated Scale of Intelligence (WASI) [33]; Controlled Oral Word Association Test (COWAT) [34]; Hayling Sentence Completion Test (HSCT) [35]; California Verbal Learning Test (CVLT) [36]; Wechsler Memory Scale immediate and delayed picture recall [37].

Neuropsychometry was divided into 3 domains: General intellectual function (full scale IQ, verbal IQ, performance IQ); Executive function (semantic and phonological verbal fluency; HSCT); Memory (CVLT total score; immediate and delayed recall on WMS). For the VPT group, Z scores of individual tests were computed, taking the Term group mean and standard deviation as reference. Within the Executive and Memory domains, Z scores were summed to create a global score which was then used in subsequent correlational analyses.

### Neonatal Cerebral Ultrasound Classification

Ultrasound (US) ratings were performed on the VPT group in the perinatal period, using a linear US array. Participants were divided into 3 groups on the basis of these ratings (0 = normal; 1 = uncomplicated periventricular haemorrhage; 2 = periventricular haemorrhage and ventricular dilatation) [8], [38] (see Table 1).

**Table 1:** Demographic details, cognitive function and neuroimaging measures for VPT and Term groups.

	Term	VPT	*Statistics*
Gender F/M	22/27	39/41	χ^2^ = 0.181; p = 0.671
Age (years)	18.6 (0.93)	19.2 (0.96)	F = 14.3; p = 0.001
**Socioeconomic status**			
I	6	5	χ^2^ = 13.03; p = 0.011
II	21	23	
III	13	45	
IV	5	6	
V	4	1	
Neonatal			
Gestational age (weeks)	40.2 (1.49)	28.9 (2.18)	F = 878.5; p<0.0005
Birth weight (g)	3317.9 (385.3)	1263.6 (393.9)	F = 710.5; p<0.0005
Length of apnoea (min)	*NA*	2.02 (2.22)	NA
Neonatal pH	*NA*	7.24 (0.11)	NA
**General intellectual functioning**			
Full-scale IQ	106.3 (13.1)	95.5 (13.9)	F = 18.5; p<0.0005
Verbal IQ	102.9 (13.8)	93.6 (14.9)	F = 12.1; p = 0.001
Performance IQ	106.7 (15.0)	98.1 (14.9)	F = 9.9; p = 0.002
**Executive Function**			
Phonological VF	41.4 (11.1)	36.3 (10.7)	F = 6.51; p = 0.012
Semantic VF	22.5 (5.0)	19.9 (5.4)	F = 7.32; p = 0.008
HSCT score	5.9 (1.5)	4.8 (2.0)	F = 11.1; p = 0.001
**Global executive score**	*Reference*	−5.7 (2.0) range [−10.1 −0.2]	
**Memory**			
CVLT total score	56.3 (8.7)	52.1 (10.4)	F = 5.59; p = 0.02
WMS immediate	11.3 (2.3)	6.9 (17.7)	F = 2.87; p = 0.093
WMS delayed	10.4 (3.0)	5.6 (17.5)	F = 3.589; p = 0.061
**Global memory score**	*Reference*	−1.8 (3.1) range [−9.2 2.5]	
**Brain volumes**			
Grey matter (cm^3^)	805.3 (84.8)	746.9 (75.2)	F = 14.3; p<0.0005
White matter (cm^3^)	455.5 (50.5)	428.7 (57.0)	F = 6.2; p = 0.014
Cerebellum (cm^3^)	140.5 (11.2)	135.8 (20.4)	F = 1.5; p = 0.222
Lateral ventricles (cm^3^)	13.1 (7.9)	20.0 (9.8)	F = 16.4; p<0.0005
Corpus callosum (mm^2^)	494.6 (98.2)	477.5 (104.5)	F = 0.624; p = 0.431

Demographic details, cognitive function and neuroimaging measures for very-preterm born and Term-born groups. Global neuropsychological scores are the sum of domain-specific *Z* scores for executive functioning and memory in the very-preterm born group. Mean and standard deviation of the Term-born group was the reference category.(Abbreviations: CVLT: California Verbal Learning Test ; HSCT: Hayling Sentence Completion Test; NA: Not assessed; NP: Neuropsychometry; Term: term born individuals; VPT: very-preterm born individuals; Wechsler Memory Scale; neonatal pH determined from cord blood at time of birth).

### Measurement of Brain Volumes

Three-dimensional T1-weighted inversion recovery prepared spoiled gradient recalled structural images (IR-SPGR) were acquired on a GE Signa 1.5 Tesla MRI system (General Electric, USA) in the same session as the DT-MRI data. The volume of the lateral ventricles was determined by the Cavalieri method, using ‘MEASURE’ (Johns Hopkins University, Baltimore, USA) [39]. Ventricular sizes were measured by a single operator. Cerebellar size was measured using similar methods, and total grey and white matter volumes were calculated using SPM5 (http://www.fil.ion.ucl.ac.uk/spm/) [40]. Corpus callosum cross-sectional area was measured using Analyze7c, following the method described in Allin et al. (2007) [31]. Using this method, the corpus callosum was divided into 4 equal sections on a mid-sagittal slice (anterior, mid-anterior, mid-posterior and posterior). Structural MRI data has been reported in detail elsewhere [31], [40].

### DT-MRI

DT-MRI images were acquired in the same session as the IR-SPGR images. The sequence provided isotropic voxels (2.5 mm×2.5 mm×2.5 mm, reconstructed as 1.875 mm×1.875 mm×2.5 mm) with coverage of the whole head, gated to the cardiac cycle, with an echo time of 107 ms, and effective repetition time of 15 R-R intervals. The duration of the diffusion encoding gradients was 17.3 ms, giving a maximum diffusion weighting of 1300 s/mm^2^ [41]. At each location, seven images without a diffusion gradient (i.e., b = 0 s/mm^2^) were acquired, along with 64 diffusion-weighted images (1300 s/mm^2^), with the latter having gradient directions distributed uniformly in space. Diffusion-weighted images were corrected for eddy-current distortions, and masked using a modified version of the brain extraction tool from the Functional Software Library (FSL) (Oxford University, UK) [42]–[43]. The method of Basser et al. (1994) [17]. was used to determine the diffusion tensor in each voxel, from which fractional anisotropy (FA) images were calculated.

### DT-MRI Processing

Group mapping techniques, derived from the computational morphometry methods developed for structural MRI were used [44], [45]. These techniques compare parametric maps calculated from MRI data (in this case, FA) between subjects or groups, following registration of each subject's map into a standard space. Initial FA images were calculated using locally written software, and registered using Statistical Parametric Mapping (SPM2, University College London, UK). A two-stage registration process was used, analogous to the ‘optimised voxel-based-morphometry’ approach [46]. To reduce the potential for mis-registration, VPT individuals were excluded if their ventricular volume exceeded the maximum measured in the Term group (46 mm^3^).

The mean T2-weighted (b = 0) image for each subject was first registered to the SPM2 EPI template and the derived warping parameters applied to the corresponding FA image. Normalised FA images of all participants were averaged and smoothed to create a new, study-specific template, to which each subject's FA images were then re-registered. Registered FA images were also segmented (again in SPM2) to give maps of the probability of a tissue being either white or grey matter, and these segmented images were thresholded at a low (10%) probability to provide a binary mask of white matter. (An accurate segmentation was not essential, and a deliberately relatively liberal threshold was used, in order to create a slightly ‘over inclusive’ mask). Next, the images were smoothed with a 5×5×5 Gaussian kernel to minimise residual mis-registration. Finally, the smoothed images were multiplied by the binary mask, restricting subsequent analyses to white matter only.

Between-group differences in FA were estimated by fitting an analysis of covariance (ANCOVA) model at each intracerebral voxel in standard space. This model examined group differences between VPT and Term subjects, using permutation based testing, implemented in the XBAM package (developed at The Institute of Psychiatry, London, UK http://www.brainmap.co.uk/) to assess statistical significance at both the voxel and cluster levels [47]. Initially, a relatively lenient p-value (p≤0.05) was set to detect voxels putatively demonstrating differences between groups. At this stage, we considered only those voxels at which all subjects contributed data. Along with the masking procedure described above, this restricts the analysis to core white matter regions, reducing the search volume (and thus the number of comparisons made) and also avoiding testing at the grey/white interfaces, where the high grey/white contrast of FA images exacerbates any edge effects. The programme then searched for spatial clusters among these voxels, and tested the ‘mass’ of each cluster (the sum of suprathreshold voxel statistics it comprises) for significance. At the cluster level the number of clusters which would be expected by chance alone for a range of p-values was calculated and used to set the statistical threshold for significance for each analysis so that the expected number of false positive clusters would be less than one. On the resulting cluster maps, affected white matter tracts were identified by reference to an atlas [48]. Mean FA values were also extracted, for each individual, from each cluster in the VPT/Term analysis, and used to test for associations between FA differences and cognitive function or (in the VPT group alone) perinatal variables.

### Statistical Analysis

Socio-demographic characteristics were analysed with χ^2^ tests (with Yates or Fisher's correction), and independent samples t-tests, as appropriate. Differences in IQ were analysed with Analysis of Covariance (ANCOVA), adjusting for the age at assessment. Relationships between FA and perinatal variables and between FA and neuropsychological function were assessed using Kendall partial correlations, controlling for age at assessment and socioeconomic status.

### Ethics Statement

Ethical approval was obtained from the Joint South London and Maudsley and the Institute of Psychiatry NHS Research Ethics Committee. All participants gave written, informed consent.

## Results

### Characteristics of the Study Groups

DT-MRI data were successfully acquired on 87 VPT and 49 Term individuals. Data from seven VPT individuals who had lateral ventricular volumes above 46 mm^3^, were excluded. Socio-demographic and cognitive characteristics of the two groups are shown in Table 1. VPT and Term groups did not differ in gender distribution, but did differ in distribution of SES, and the VPT group was slightly, but significantly, older than the Term group at assessment (Table 1). Subsequent analyses were corrected for age and SES.

### Cognitive Function

The VPT group had significantly lower full-scale, verbal and performance IQ than the Term group. Total CVLT score, HSCT score, and semantic and phonological verbal fluency were significantly lower in the VPT group. There was no significant group difference in WMS (Table 1).

### Structural Brain Measurements

Lateral ventricular volume was significantly increased in the VPT group – even after exclusion of 7 individuals with extreme ventricular enlargement. Total grey and white matter volumes and corpus callosum cross-sectional area were significantly reduced in the VPT group. There was no significant group difference in cerebellar volume or corpus callosum cross-sectional area (Table 1).

### Group Differences in FA

**Figure 1:**
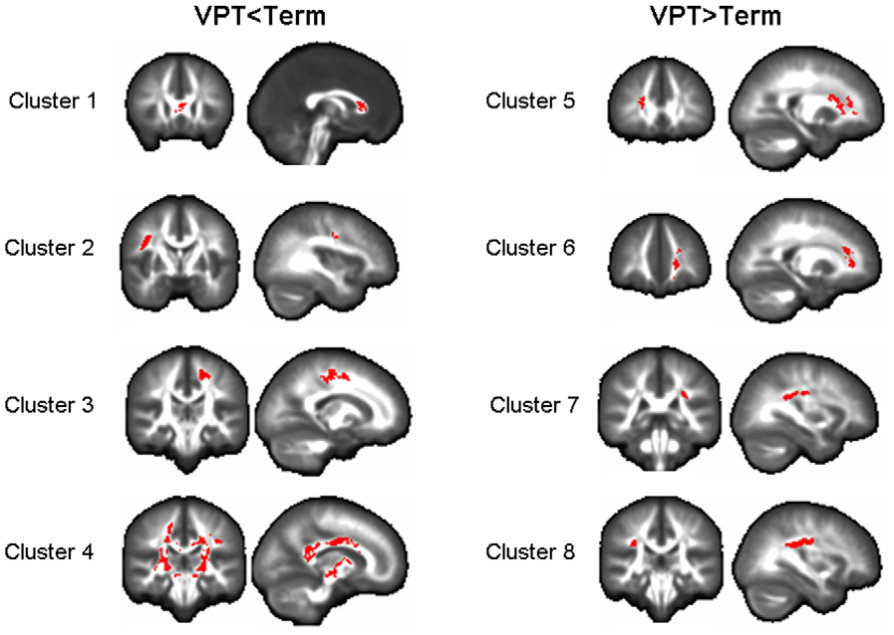
Group differences in FA, displayed on a white matter template. Group differences in fractional anisotropy, displayed on representative white matter template using MRIcro (http://www.cabiatl.com/mricro/). One sagittal and one coronal view is displayed for each cluster showing significant group differences. Cluster numbers refer to Table 1, where MNI coordinates and tract identification are given.

FA was reduced in the VPT group relative to the Term group in four clusters, containing a total of 4688 voxels (p = 0.0075). While our voxel based approach provides results relating to regions of the brain, not white matter tracts per se, comparison of these regions with atlases [48] suggests that the differences we see fall largely within: corpus callosum (genu, splenium and body); left and right superior longitudinal fasciculi; left superior corona radiata; left and right superior longitudinal fasciculi (See Table 2 and Figure 1).

**Table 2:** Group differences in Fractional Anisotropy (FA).

Number of Voxels	Cluster ID	White Matter Regions and Tracts	MNI Coordinates			p
		VPT<Term	x	y	z	
157	1	Corpus callosum (genu)	0	26	4	0.0009
108	2	R Superior longitudinal fasciculus	45	−2	16	0.001
154	3	L Superior corona radiata	−18	−20	50	0.0004
4269	4	Corpus callosum (body and splenium); L&R Superior longitudinal fasciculi	14	−37	6	0.0001

		VPT>Term	x	y	z	
153	5	R Inferior fronto-occipital and uncinate fasciculi; anterior corona radiata	22	30	8	0.002
214	6	L Inferior fronto-occipital and uncinate fasciculi; anterior corona radiata	−18	35	0	0.0001
143	7	L Superior longitudinal fasciculus	−25	−35	24	0.0001
286	8	R Superior longitudinal fasciculus	31	−33	22	0.0001

Group differences in Fractional Anisotropy (FA). The co-ordinates of the centres of mass of significant clusters are given, along with their anatomical (tract) location. (Abbreviations: L: left; MNI: Montreal Neurological Institute; R: Right; Term: term-born individuals VPT: very-preterm born individuals).

Additionally, there were four clusters (containing a total of 796 voxels; p = 0.0075) in which FA was higher in the VPT group than in the Term group. These clusters involved regions likely to represent: left and right inferior fronto-occipital fasciculus; left and right uncinate fasciculi; left and right superior longitudinal fasciculi; left and right anterior corona radiata.

### Relationships Between Perinatal Variables and FA

Mean FA values were extracted from the clusters in the group maps, and relationships with other variables assessed using partial correlations (controlling for age at assessment and SES). This analysis was restricted to the VPT group (perinatal data were not available for the Term group). FA in cluster 2, corresponding to the right superior longitudinal fasciculus (VPT<Term), was positively associated with gestational age and birth weight. FA in cluster 4, localised to the body and splenium of corpus callosum and bilateral superior longitudinal fasciculus (VPT<Term), was associated with birth weight, but not gestational age. Of the VPT>Term clusters, FA in clusters 5 and 6 (corresponding to the right and left inferior fronto-occipital and uncinate fasciculi and right and left anterior corona radiata) was negatively associated with gestational age (increasing gestational age associated with lower FA). FA in cluster 8, localised to right superior longitudinal fasciculus (VPT>Term), was positively associated with gestational age and birth weight (higher gestational age or birth weight associated with higher FA) (Table 3). There was no statistically significant association between FA and indicators of neonatal hypoxia (neonatal pH; length of apnoea). ANOVA comparing neonatal ultrasound categories revealed significant differences in all 4 VPT>Term clusters, where FA was significantly higher in the uncomplicated periventricular haemorrhage and the periventricular haemorrhage plus dilatation groups (Table 4). There were no ultrasound severity group differences in the VPT<Term clusters.

**Table 3:** Correlations between FA and perinatal, neuropsychological and neuroimaging variables.

	FA cluster							
	VPT<Term				VPT>Term			
	1	2	3	4	5	6	7	8
**Gestational age (weeks)**	NS	0.240 p = 0.034	NS	NS	−0.277 p = 0.014	−0.233 p = 0.040	NS	0.240 p = 0.034
**Birth Weight (g)**	NS	0.406 p<0.0005	NS	0.293 p = 0.009	NS	NS	NS	0.406 p<.0005
**fsIQ**	0.273 p = 0.027	0.315 p = 0.010	NS	NS	NS	NS	NS	NS
**vIQ**	NS	NS	NS	NS	NS	NS	NS	NS
**pIQ**	0.359 p = 0.003	0.416 p = 0.001	0.238 p = 0.055	0.320 p = 0.009	NS	NS	NS	NS
**Executive function**	NS	NS	NS	NS	NS	NS	NS	NS
**Memory**	0.253 p = 0.040	0.403 p = 0.001	0.326 p = 0.007	0.271 p = 0.028	NS	NS	NS	NS
**Grey matter (cm^3^)**	NS	NS	NS	NS	NS	NS	NS	NS
**White matter (cm^3^)**	0.314 p = 0.021	NS	NS	NS	NS	NS	NS	NS
**Cerebellum (cm^3^)**	NS	NS	NS	NS	NS	NS	NS	NS
**Lateral ventricles (cm^3^)**	−0.455 p = 0.001	NS	−0.433 p = 0.001	−0.703 p<0.0005	NS	NS	NS	NS
**Corpus callosum (mm^2^)**	0.334 p = 0.014	0.362 p = 0.007	NS	0.384 p = 0.004	NS	NS	NS	NS

Partial correlation coefficients (controlling for age at assessment and socioeconomic status) between cluster mean fractional anisotropy values and perinatal, neuropsychological and neuroimaging variables in the very-preterm born group only. Cluster numbers refer to Figure 1 and Table 2. (Abbreviations: NP = Neuropsychometry; MRI = Magnetic Resonance Imaging; NS = not significant (p>0.05)).

**Table 4:** FA in relation to severity of neonatal brain injury in the VPT group.

		Neonatal ultrasound abnormality			ANOVA
Cluster	Location	None	PVH	PHV+dil	
5	R IFO, uncinate	0.37 (0.3)	0.39 (0.2)	0.39 (0.3)	F = 4.12; df = 58; p = 0.021
6	L IFO, uncinate	0.38 (0.3)	0.40 (0.2)	0.41 (0.1)	F = 6.22; df = 58; p = 0.004
7	L SLF	0.40 (0.3)	0.41 (0.3)	0.44 (0.2)	F = 4.63; df = 58; p = 0.014
8	R SLF	0.43 (0.3)	0.44 (0.2)	0.46 (0.2)	F = 5.00; df = 58; p = 0.010

Fractional anisotropy (FA) [mean (SD)] of centres of clusters showing group differences (Table 2; Figure 1), divided according to severity of brain injury on neonatal ultrasound in the VPT group. Abbreviations: PVH = uncomplicated periventricular haemorrhage; PHV+dil = periventricular haemorrhage plus dilatation ; IFO = Inferior Fronto-Occipital fasciculus; L: left; SLF = Superior Longitudinal Fasciculus; PCR = Posterior Corona Radiata; R:right; Term: term- born individuals; VPT: very-preterm born individuals.

NS = p>0.05.

### Relationships Between Cognitive Functioning and FA

There were no associations between FA and neuropsychometry in the Term group. In the VPT group, significant associations between FA and performance IQ and between FA and Memory were found in all 4 of the VPT<Term clusters, such that higher FA was associated with better cognitive function (Table 3). Similarly, full scale IQ was associated with FA in clusters 1 and 2. There were no associations between FA and verbal IQ, or FA and Executive Function in any cluster.

### Relationships Between Adult Neuroimaging Data and FA

Lateral ventricular volume was negatively associated with FA in 3 VPT<Term clusters 1, 3 and 4. Corpus callosum cross sectional area was positively associated with FA in VPT<Term clusters 1, 2 and 4. There were no significant associations between total grey or white matter volume or cerebellar volume in any FA cluster (Table 3). There were no associations between FA and structural brain measures in the Term group.

## Discussion

We have demonstrated spatially widespread alterations in white matter microstructure in VPT young adults, affecting areas likely to represent the corpus callosum, sensorimotor tracts and many long association tracts in both hemispheres. These microstructural alterations are associated with birth weight and gestational age, and with adult cognitive function.

Our results are consistent with other studies that have examined FA in VPT individuals at different ages – microstructural white matter abnormalities have been reported in preterm or low birth weight neonates [25], infants [49], children [21], [24], and adolescents [50]. For example, Constable et al. (2008) [51] demonstrated FA reductions in inferior fronto-occipital fasciculus, anterior uncinate and splenium of the corpus callosum in preterm 12-year-olds. Similar patterns of FA reductions have been reported in VLBW adolescents, affecting the internal capsule, corpus callosum, and hemispheric association tracts [29], [30]. Eikenes et al. (2010) [52] have studied FA and mean diffusivity (MD) in very-low-birth-weight (VLBW≤1500 g) adults at a comparable age to our sample (18–22 years). They also found decreased FA in many regions/tracts, including the corpus callosum. Similarly, Mullen et al. (2011) [53] have demonstrated reduced FA in preterm 16-year-olds in several regions, including uncinate fasciculus, external capsule, corpus callosum (splenium), and frontal white matter.

Our findings differ from other studies in that we found clusters where FA was increased in the VPT group relative to the Term group. This was unexpected, and merits further discussion. Similar FA increases were reported by Vangberg et al. (2006) [29] in very-low-birth-weight adolescents and by Eikenes et al. (2010) [52], although not by Skranes et al. (2007) [30] in a similar sample. Methodological differences may underlie this discrepancy. First, Skranes et al. (2007) [30] studied individuals selected by birth weight rather than gestational age – and although overlapping, these two populations are not identical. Second, their participants were scanned at a younger age than ours. There is active growth of white matter and increase in FA during adolescence [54], [55]. In a previous study, we have shown a striking pattern of increased growth of the corpus callosum between adolescence and adulthood in VPT individuals [31]. Differential growth of white matter between VPT and term groups could alter the pattern of group differences that are observed at different age-ranges. In a comparably-aged group of very-low-birth-weight adults, Eikenes et al (2010) [52] reported one area of increased FA in the very-low-birth-weight group relative to controls, which is consistent with our findings. In our study, the areas of increased FA in the VPT group were relatively small (796 voxels) compared to the areas of FA decrease (4688 voxels) – a similar pattern to that reported by Eikenes et al (2010) [52]. Given the consistency of this finding across studies, and the relationships that we have demonstrated between these areas of FA increase and neonatal brain injury, we suggest that areas of increased FA in VPT and very-low-birth-weight adults may be biologically meaningful. There is some evidence that, in children and adolescents, there are negative correlations between corpus callosum thickness and cognitive function [56] – so, for white matter at least, more does not always mean better.

Regions of increased FA in the VPT group could also represent an ‘unmasking’ effect. In regions of crossing fibres, an individual voxel is likely to contain fibres of more than one orientation. The mean FA of such a voxel could be relatively low (especially if the vectors of the crossing tracts diverge significantly). If this voxel were then to lose some fibres its apparent FA could increase, as the remaining fibres would have a more ‘coherent’ mean orientation. In this model, FA increases in the VPT group relative to the Term group could actually represent regions of white matter loss in the VPT group. The exact microstructural and anatomical correlates of DT-MRI, and how they change during development, are as yet not fully known [57].

Alternatively, clusters of increased FA in the VPT group might be indicative of compensatory changes – where plasticity of white matter has allowed function to be spared, although white matter integrity has been disturbed by early brain insults. Some of our findings are compatible with this explanation: VPT participants with more severe neonatal brain injury (by ultrasound) had higher FA in several clusters; one cluster of increased FA was associated with lower gestational age (right superior longitudinal fasciculus). In a structural MRI (not DT-MRI) study, Nosarti et al. (2008) [10] demonstrated white matter increases in VPT adolescents who had experienced more severe grades of perinatal brain injury.

Two clusters of reduced FA were associated with birth weight and gestational age, although the correlations between FA and birth weight were stronger than those between FA and gestational age. The large cluster involving corpus callosum and corticospinal tract was associated with birth weight only, and the cluster involving the right superior longitudinal fasciculus was associated with both birth weight and gestational age. Although there is considerable overlap, the categories of low birth weight and preterm birth may be indicative of different pathological processes. Our results may indicate that gestational age and birth weight have differential relationships to adult white matter, but we were not able to assess patterns of intrauterine growth which would be necessary properly to address this question. Andrews et al. (2010) [58] demonstrated a relationship between birth weight and corpus callosum FA in preterm children (mean age at assessment 11 years). Eikenes et al. (2010) [52] in very-low-birth-weight young adults also showed relationships between FA reduction and perinatal adversity, including gestational age, birth weight, days in Neonatal Intensive Care and length of mechanical ventilation. White matter injury is common in VPT and very-low-birth-weight neonates, and its prevalence and severity is related to perinatal adversity [6].

We also have demonstrated associations between structural neuroimaging measures and FA in several clusters. These clusters were all ones in which FA was reduced in the VPT group. Notably, corpus callosum size was associated with FA in clusters 1, 2 and 4. Two of these clusters anatomically involved the corpus callosum. Thus the structural MRI findings are consistent with the DT-MRI findings. Bassi et al (2011) [69] report relationships between white matter lesions and reduced FA in corticospinal tracts or preterm infants, which is consistent with our findings. Lateral ventricular volume was also associated with FA in 3 of the VPT<Term clusters. Enlargement of the lateral ventricles is a known sequel of perinatal brain injury in VPT infants [59] and is associated with white matter damage, and subsequently reduced white matter volumes [8].

Alterations of FA in the VPT group were also associated with cognitive outcome. IQ was associated with FA in three of the VPT<Term clusters. This relationship was specific for performance IQ rather than verbal IQ. We also found significant associations between higher FA and better global memory function, in all 4 clusters of reduced FA. Associations between FA and cognition have been observed in other VPT and very-low-birth-weight groups, and our results are consistent with this [22], [30]. The lack of an association between verbal IQ and FA is striking, particularly given that several of the tracts involved (notably the superior longitudinal fasciculus) are part of the anatomy underlying language function. Successful performance on the performance IQ subtests of the WASI requires an element of timed performance, with bimanual coordination and spatial reasoning (the block design subtest). This requires the coordinated action of several different brain areas, and is likely to be dependent on intact and functioning white matter. In a parallel study using DT-MRI tractography in this same group of VPT adults Kontis et al. (2009) [31] showed that altered microstructure in the genu of the corpus callosum is associated with lower performance IQ. Andrews et al. (2010) [58] found that reading skill in 11 year old preterm children was associated with FA of the genu and body of the corpus callosum. The pattern of association of IQ with multiple white matter regions or tracts has face-validity, since IQ is a composite of multiple cognitive processes [60] associated with the structure and function of several connected brain regions [61]. Our results are consistent with the concept that the distributed neural networks underlying cognition are altered in VPT adults. Consistent results are reported by Mullen at al. (2011) [53], who demonstrated correlations between uncinate fasciculus FA a semantic language task, and between arcuate fasciculus FA and a phonological task. Like us, Mullen et al. (2011) [53] found these relationships only in their preterm participants, and not in their term-born control group. They suggest that their findings indicate that neural networks are altered in preterm individuals, or that this represents delayed maturation in the preterm group relative to the control group. Our findings are also consistent with these explanations. It is of interest is that correlations with IQ and memory in our study were only found in regions of FA reduction in the VPT group, and not in regions where FA was increased. A similar pattern of association between white matter volume (not DT-MRI) and cognition was reported by Nosarti et al (2008) [10]. This would be consistent with the suggestion that areas of increased FA in the VPT group represent compensatory changes. Lubsen et al. (2011) [62] have suggested that neurodevelopmental sequelae of preterm birth are due to altered patterns of neural connectivity. Evidence from functional MRI studies [63]–[65] indicates that VPT adolescents and adults have altered neural networks underlying a variety of cognitive domains. The functional connectivity study of Myers et al. (2010) [66] is also consistent with this concept.

We acknowledge a number of possible limitations in the interpretation of these results. First, co-registration of low-resolution, high-contrast FA maps may give rise to mis-registration and partial volume artefacts in regions of high and low anisotropy, for example, around the ventricles. In order to minimise such artifacts we used a two-step registration process and a masking procedure which restricted analyses to core white matter regions; we also excluded VPT participants with ventriculomegaly, in whom any such issues are likely to be exacerbated. Second, resolving a cluster into component tracts by reference to anatomical atlases is compromised by the limited resolution of the parametric maps and by the limited white matter detail such atlases contain. Third, the correlation analyses that we report could be vulnerable to type I errors by virtue of the number of comparisons made. Fourth, there are systematic differences between our participant groups, including an age difference between VPT and term comparison groups. Since FA is known to change with age, this could have introduced bias into our results. We have attempted to adjust statistically for this possibility in all the analyses.

There is much still to discover about the lifespan development of white matter after premature birth. Longitudinal studies remain the best method of addressing this kind of question, although they are not easy to carry out, and require commitment from funding bodies and research institutions if they are to be sustained for the length of time required. A recently-published MRI atlas of neonatal brain development was able to image maturational changes in the neonatal period in normally developing babies [70]. Such methodology could provide information about brain development after preterm birth, and may be able to identify plausible sensitive developmental periods during which to target therapeutic interventions. The interaction between brain lesions, development and social and economic factors are not yet well studied. Nagy et al. (2009) [73] found milder-than-expected brain abnormalities in very preterm adolescents born in the late 1980s and early 1990s, and speculate that such factors may be important in changing brain structural outcomes. New imaging techniques may also prove informative. For example, Driven Equilibrium Single Pulse Observation of T1 and T2 (quaintly known as “DESPOT”) can now be used to estimate characteristics such as myelination [68]. Such emerging techniques have the potential to tell us more about the pathological and developmental processes affecting white matter after VPT birth.

### Conclusion

VPT young adults have widespread alterations of fractional anisotropy, which are related to gestational age and birth weight. Some microstructural alterations may represent plastic reorganisation of white matter as well as the effects of early brain lesions. White matter microstructure is associated with cognitive outcome.
